# *Ochrobactrum quorumnocens* sp. nov., a quorum quenching bacterium from the potato rhizosphere, and comparative genome analysis with related type strains

**DOI:** 10.1371/journal.pone.0210874

**Published:** 2019-01-22

**Authors:** Dorota M. Krzyżanowska, Tomasz Maciąg, Adam Ossowicki, Magdalena Rajewska, Zbigniew Kaczyński, Małgorzata Czerwicka, Łukasz Rąbalski, Paulina Czaplewska, Sylwia Jafra

**Affiliations:** 1 Laboratory of Biological Plant Protection, Intercollegiate Faculty of Biotechnology of University of Gdansk and Medical University of Gdansk, University of Gdansk, Gdansk, Poland; 2 Laboratory of Structural Biochemistry, Faculty of Chemistry, University of Gdansk, Gdansk, Poland; 3 Laboratory of Recombinant Vaccines, Intercollegiate Faculty of Biotechnology of University of Gdansk and Medical University of Gdansk, University of Gdansk, Gdansk, Poland; 4 Laboratory of Mass Spectrometry, Intercollegiate Faculty of Biotechnology of University of Gdansk and Medical University of Gdansk, University of Gdansk, Gdansk, Poland; Institut National de la Recherche Agronomique, FRANCE

## Abstract

*Ochrobactrum* spp. are ubiquitous bacteria attracting growing attention as important members of microbiomes of plants and nematodes and as a source of enzymes for biotechnology. Strain *Ochrobactrum* sp. A44^T^ was isolated from the rhizosphere of a field-grown potato in Gelderland, the Netherlands. The strain can interfere with quorum sensing (QS) of Gram-negative bacteria through inactivation of *N*-acyl homoserine lactones (AHLs) and protect plant tissue against soft rot pathogens, the virulence of which is governed by QS. Phylogenetic analysis based on 16S rRNA gene alone and concatenation of 16S rRNA gene and MLSA genes (*groEL* and *gyrB*) revealed that the closest relatives of A44^T^ are *O*. *grignonense* OgA9a^T^, *O*. *thiophenivorans* DSM 7216^T^, *O*. *pseudogrignonense* CCUG 30717^T^, *O*. *pituitosum* CCUG 50899^T^, and *O*. *rhizosphaerae* PR17^T^. Genomes of all six type strains were sequenced, significantly expanding the possibility of genome-based analyses in *Ochrobactrum* spp. Average nucleotide identity (ANIb) and genome-to-genome distance (GGDC) values for A44^T^ and the related strains were below the single species thresholds (95% and 70%, respectively), with the highest scores obtained for *O*. *pituitosum* CCUG 50899^T^ (87.31%; 35.6%), *O*. *rhizosphaerae* PR17^T^ (86.80%; 34.3%), and *O*. *grignonense* OgA9a^T^ (86.30%; 33.6%). Distinction of A44^T^ from the related type strains was supported by chemotaxonomic and biochemical analyses. Comparative genomics revealed that the core genome for the newly sequenced strains comprises 2731 genes, constituting 50–66% of each individual genome. Through phenotype-to-genotype study, we found that the non-motile strain *O*. *thiophenivorans* DSM 7216^T^ lacks a cluster of genes related to flagella formation. Moreover, we explored the genetic background of distinct urease activity among the strains. Here, we propose to establish a novel species *Ochrobactrum quorumnocens*, with A44^T^ as the type strain (= LMG 30544^T^ = PCM 2957^T^).

## Introduction

**Fig 1:**
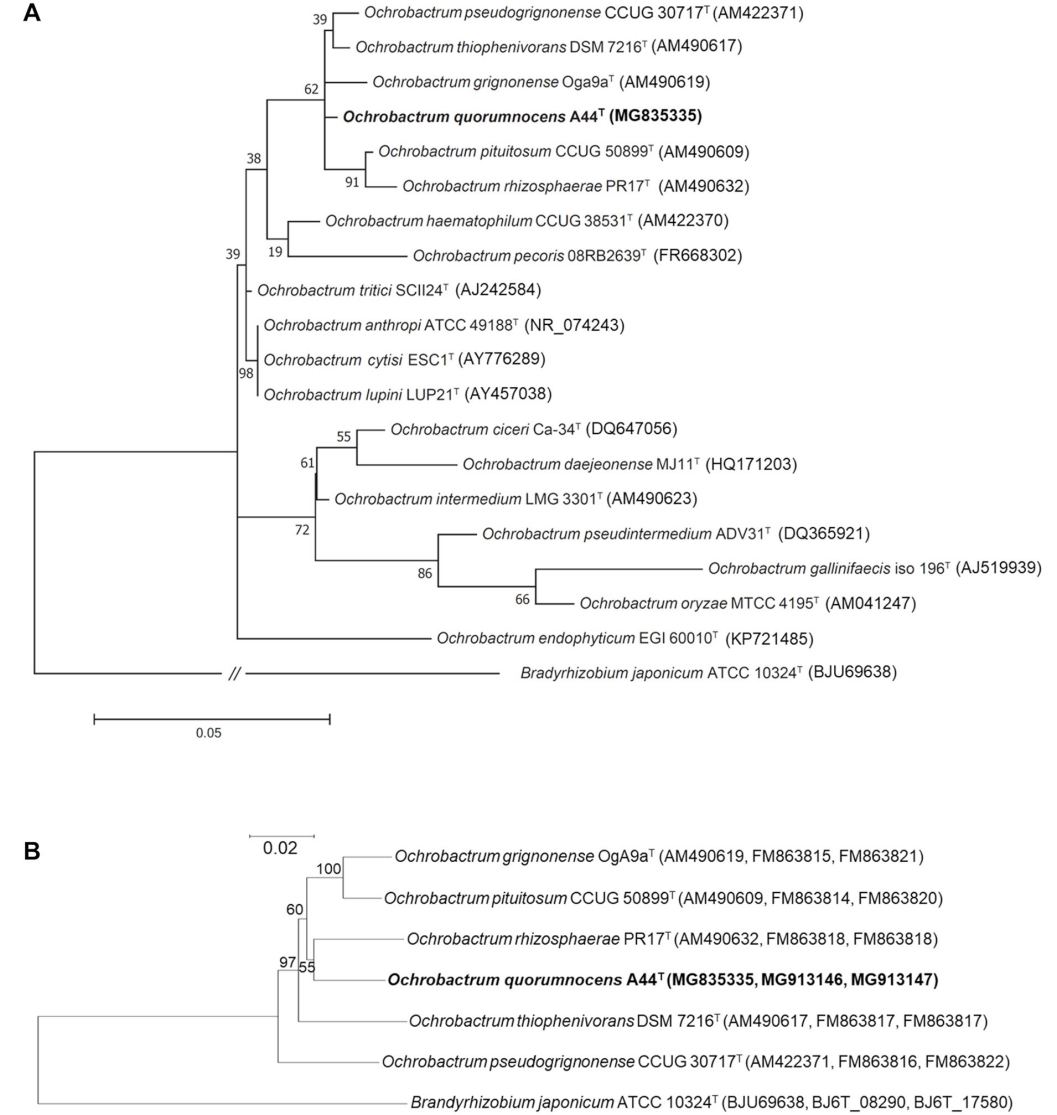
Phylogenetic trees depicting the phylogenetic position of *Ochrobactrum* sp. A44^T^ strain among other members of the *Ochrobactrum* genus. (A) Molecular phylogenetic analysis of partial (1335 bp) 16S rRNA gene sequences by the maximum likelihood method based on the Tamura 3-parameter model. Analyses were conducted in MEGA7. The tree with the highest log likelihood (-3900.57) is shown. The percentage of trees in which the associated taxa clustered together is shown next to the branches. (B) Dendrogram based on MLSA performed on concatenated nucleotide sequences of three genes: 16S rRNA gene (1335 bp), *groEL* (1165 bp), and *gyrB* (1012 bp). The tree was obtained using the neighbor-joining algorithm. Bootstrap values based on 1000 replicates are shown at the nodes. *Bradyrhizobium japonicum* ATCC 10324^T^ was used as an outgroup. Bar indicates number of substitutions per site. Accession numbers of gene sequences are shown in brackets.

*Ochrobactrum* spp., together with the closely related *Brucella*, *Agrobacterium*, and *Rhizobium* genera, belong to the class of *Alphaproteobacteria* [1–3]. Although the genus is most often associated with *O*. *anthropi* [4] and *O*. *intermedium* [1], which cause opportunistic infections in humans, the bacteria from the *Ochrobactrum* spp. genus adapted to a variety of environmental niches and can be found in soil [5], wastewater [6], in association with plants [6,7], and animals [8,9]. The ability of *Ochrobactrum* spp. members to utilize xenobiotic compounds led to their exploration as potential bioremediation agents [10–13] or a source of enzymes for the biotech industry [14,15]. *Ochrobactrum* spp. are also of interest as plant beneficial bacteria [16,17]. The plant-derived strains, such as *O*. *lupini* LUP21^T^ [7] and *O*. *cytisi* ESC1^T^ [18], are able to nodulate roots to fix nitrogen, underlining their close association with the host plants. Currently, the *Ochrobactrum* genus comprises 18 species [19] (Fig 1A). However, scientific data concerning the majority of the strains is limited to information of taxonomic value.

A44^T^ is a potato rhizosphere isolate, obtained in 2002 from the experimental field in Bennekom, the Netherlands, and studied for its ability to inactivate *N*-acyl homoserine lactones (AHLs) [20] *via* AHL-acylase activity [21]. The AHLs are small molecules produced, secreted, and auto-sensed by many Gram-negative bacteria in a regulatory mechanism called quorum sensing (QS). In QS, the expression of target genes is modulated in the presence of a threshold concentration of signal molecules in the immediate environment of the cell [22,23]. QS is known to govern vital metabolic processes in the AHL-utilizing bacteria, including production of virulence factors and biofilm formation. This enables strains like A44^T^ to deplete the pool of AHLs and interfere with QS of other species of scientific and applicational interest [24,25]. It was shown that through inactivation of AHLs, A44^T^ is able to attenuate the QS-dependent virulence of *Pectobacterium parmentieri* SCC3193 [26] and *P*. *carotovorum* EC71 [27] and hence protect the plant tissue from soft rot caused by these pathogens [20].

In this study, we establish the taxonomic position of *Ochrobactrum* sp. strain A44^T^ using sequence-based, genome-scale calculations, supported by the analysis of chemotaxonomic markers and phenotypic assays. Based on the results, we propose delineation of a novel species *Ochrobactrum quorumnocens* sp. nov. with A44^T^ (= LMG 30544^T^ = PCM 2957^T^) as a type strain. Furthermore, we perform comparative genome analysis of A44^T^ and the related *Ochrobactrum* spp. and use the genomic data to link chosen differential phenotypes for the studied group to their genetic background. The only study involving genetic analysis of *Ochrobactrum* spp. concerns variation among isolates of *Ochrobactrum intermedium* and was presented by Aujoulat *et al*. [28] and Kulkarmi *et al*. [29]. To our knowledge, this is the first attempt of such analyses for other members of this genus.

## Results and discussion

### 16S rRNA gene analysis and MLSA

First, we performed phylogenetic analysis of A44^T^ and the type strains of all 18 validly published *Ochrobactrum* spp. (Fig 1A; S1A and S1B Fig). Based on 16S rRNA gene sequence analysis, the closest relatives of A44^T^ were *O*. *thiophenivorans* DSM 7216^T^, isolated from wastewater in Germany [6], *O*. *pseudogrignonense* CCUG 30717^T^, isolated from a blood sample in Sweden [30], *O*. *grignonense* OgA9a^T^, obtained from bulk soil in France [5,30], *O*. *rhizosphaerae* PR17^T^, originating from the roots of potato grown in Austria [6], and *O*. *pituitosum* CCUG 50899^T^, isolated from industrial environment in Sweden [31] (Fig 1A; S1 Table). It was already shown that the listed A44^T^-related strains cluster on a separate branch of the phylogenetic tree of *Ochrobactrum* spp. [30]. To obtain higher phylogenetic resolution within this group, we employed Multilocus Sequence Analysis (MLSA) [32] based on the concatenated sequences of 16S rRNA, *gyrB*, and *groEL* genes, which suggest that the closest relative of A44^T^ is *O*. *rhizosphaerae* PR17^T^ (Fig 1B).

### Genome sequencing and genome-based phylogeny

At the time of this study, genome sequences of only two out of eighteen *Ochrobactrum* sp. type strains were publicly available, namely *O*. *anthropi* ATCC 49188^T^ (CP000758.1-CP000763.1) and *O*. *intermedium* LMG 3301^T^ (ACQA00000000.1), thereby limiting the application of the whole-genome resolution tools in *Ochrobactrum* taxonomy, as well as comparative genomics. Hence, we obtained the genome sequences of A44^T^ and the five closely related type strains of the *Ochrobactrum* genus: *O*. *grignonense* OgA9a^T^, *O*. *thiophenivorans* DSM 7216^T^, *O*. *pseudogrignonense* CCUG 30717^T^, *O*. *pituitosum* CCUG 50899^T^, and *O*. *rhizosphaerae* PR17^T^. Complete genome sequence of A44^T^ (DDBJ/ENA/GenBank accessions of four replicons: CP022602.1-CP022605.1) was obtained by hybrid assembly of data from Illumina HiSeq2500 and PacBio RS (BaseClear B.V., the Netherlands). Draft genome sequences of *O*. *rhizosphaerae* PR17^T^ (NNRK00000000.1), *O*. *thiophenivorans* DSM 7216^T^ (NNRJ00000000.1), *O*. *grignonense* OgA9a^T^ (NNRL00000000.1), and *O*. *pseudogrignonense* CCUG 30717^T^ (NNRM00000000.1) were obtained using Illumina HiSeq2500, and the draft genome sequence of *O*. *pituitosum* CCUG 50899^T^ (PYSY00000000.2) was generated with the use of a combination of Illumina MiniSeq and Oxford Nanopore MinION platforms. With the exception of *O*. *pituitosum* CCUG 50899^T^, all sequences were annotated using the IGS Annotation Engine (Institute for Genome Sciences, University of Maryland School of Medicine, USA) [33]. Basic features of the obtained genomes are presented in S2 Table.

The complete genome of A44^T^ consists of four replicons: the main chromosome (2585.393 kbp; CP022604.1), with classical replication system, a chromid (2008.185 kbp; CP022603.1) carrying a plasmid-type replication system and the genes essential for the basic metabolism [34], [35], and two plasmids pOqn1 (1032.012 kbp; CP022605.1) and pOqn2 (19.701 kbp; CP022602.1). Genomes comprising several replicons were also reported for *O*. *anthropi* ATCC 49188^T^ [36] and the non-type *O*. *pituitosum* strain AA2 [37]. Interestingly, pOqn2 from A44^T^, as well as the pOAN03 from *O*. *anthropi* ATCC 49188^T^ [36], lacks the typical replication/partition plasmid characteristics. Presence of multiple replicons, including chromids and megaplasmids, is widespread among the members of *Alphaproteobacteria* [35].

Out of the total of 5272 open reading frames comprising the genome of A44^T^, 3329 (57.3%) have assigned function, 1443 (24.9%) were considered conserved hypothetical, and the remaining were either of unknown function: 546 (9.4%) or unclassified: 480 (8.3%). Detailed role category breakdown for all ORFs of A44^T^ genome is given in the S4 Table. Metabolic maps created for the strains are available from the Kyoto Encyclopedia of Genes and Genomes (KEGG) (https://www.genome.jp/kegg/) [38], organism number T05040.

We performed a whole-genome phylogenetic analysis for A44^T^, the other five newly-sequenced strains, and the type strains of *O*. *anthropi* and *O*. *intermedium*. Highest similarity scores based on ANIb and Genome-to-Genome Distance Calculator 2.0 (GGDC) were obtained for *O*. *pituitosum* CCUG 50899^T^ (ANIb = 87.31%, GGDC = 35.6%), followed by *O*. *rhizosphaerae* PR17^T^ (ANIb = 86.80%, GGDC = 34.3%), *O*. *grignonense* OgA9a^T^ (ANIb = 86.30%, GGDC = 33.6%), *O*. *pseudogrignonense* CCUG 30717^T^ (ANIb = 82.23%, GGDC = 26.5%), *O*. *thiophenivorans* DSM 7216^T^ (ANIb = 81.04%, GGDC = 25.3%), *O*. *anthropi* ATCC 49188^T^ (ANIb = 77.51%, GGDC = 23%), and *O*. *intermedium* LMG 3301^T^ (ANIb = 77.26%, GGDC = 22.5%) (Table 1). None of the obtained values exceeded the single species thresholds recommended for ANIb (95%) and GGDC (70%), indicating that A44^T^ should be classified as a novel species. For comparison, ANIb and GGDC values calculated for the type strains of *O*. *pituitosum* vs *O*. *grignonense* were 91.52% and 47.90%, respectively.

**Table 1:** Average nucleotide identity (ANI) values, digital DNA-DNA hybridization (*d*DDH) and G+C content (mol%) for the genome sequences of *O*. *quorumnocens* A44^T^ and the related strains.

Species	Strain	GenBank Accession	Genome assembly level (contig no.)	Sequence lenght (Mbp)	G+C (mol%)	GGDC^A^ (%)	ANIb (%)
*O*. *quorumnocens*	A44^T^	CP022602.1—CP022605.1	complete (4)	5.65	53.16	-	-
*O*. *pituitosum*	CCUG 50899^T^	PYSY00000000.2	draft (10)	5.52	53.40	**35.6**^B^	**87.31**
*O*. *rhizosphaerae*	PR17 ^T^	NNRK00000000.1	draft (36)	4.90	53.01	34.3	86.80
*O*. *grignonense*	OgA9a^T^	NNRL00000000.1	draft (169)	4.84	54.15	33.6	86.30
*O*. *pseudogrignonense*	CCUG 30717^T^	NNRM00000000.1	draft (53)	5.53	53.99	26.5	82.23
*O*. *thiophenivorans*	DSM 7216^T^	NNRJ00000000.1	draft (77)	4.36	51.65	25.3	81.04
*O*. *anthropi*	ATCC 49188^T^	CP000758.1 –CP000763.1	complete (6)	5.20	56.13	23	77.51
*O*. *intermedium*	LMG 3301^T^	ACQA00000000.1	draft (4)	4.72	57.7	22.5	77.26

^A^ genome-to-genome distance, a form of digital DNA-DNA hybridization

^B^ the highest of the calculated values are shown in bold.

Until June 2018, sixty six genome assemblies for strains identified as belonging to the *Ochrobactrum* genus were available in GenBank (NCBI). Twenty two of the sequenced strains were classified only to the genus level. Others were assigned to certain species, however, most of them without documented phylogenetic studies. We used JSpeciesWS (http://jspecies.ribohost.com/jspeciesws/) to calculate ANIb value between the genome of A44^T^ and all 65 assemblies in order to find other strains potentially representing *O*. *quorumnocens* sp. nov. No other representatives of *O*. *quorumnocens* were found based on this search. The highest ANIb score (87.54%) was obtained between A44^T^ and strain SJY1 (AZRT01000000), designated as *O*. *rhizosphaerae* (S3 Table). Interestingly, the A44^T^ is also very closely related (ANIb>87%) to a set of *Ochrobactrum* spp. isolates recently obtained from the nematode *Caenorhabditis elegans* dwelling on a rotten apple (S3 Table). Data obtained in this work can significantly facilitate assigning those isolates to the respective species. We have also found that the ANIb value for *O*. *rhizosphaerae* SJY1 and PR17^T^, the *O*. *rhizosphaerae* type strain, is 86.10%, thus below the single species threshold. ANIb calculation for *O*. *rhizosphaerae* strain SJY1 and *O*. *pituitosum* CCUG 50899^T^ suggests that SJY1 should be classified as *O*. *pituitosum* (ANIb = 96.97%).

### Comparative genome analysis

**Fig 2:**
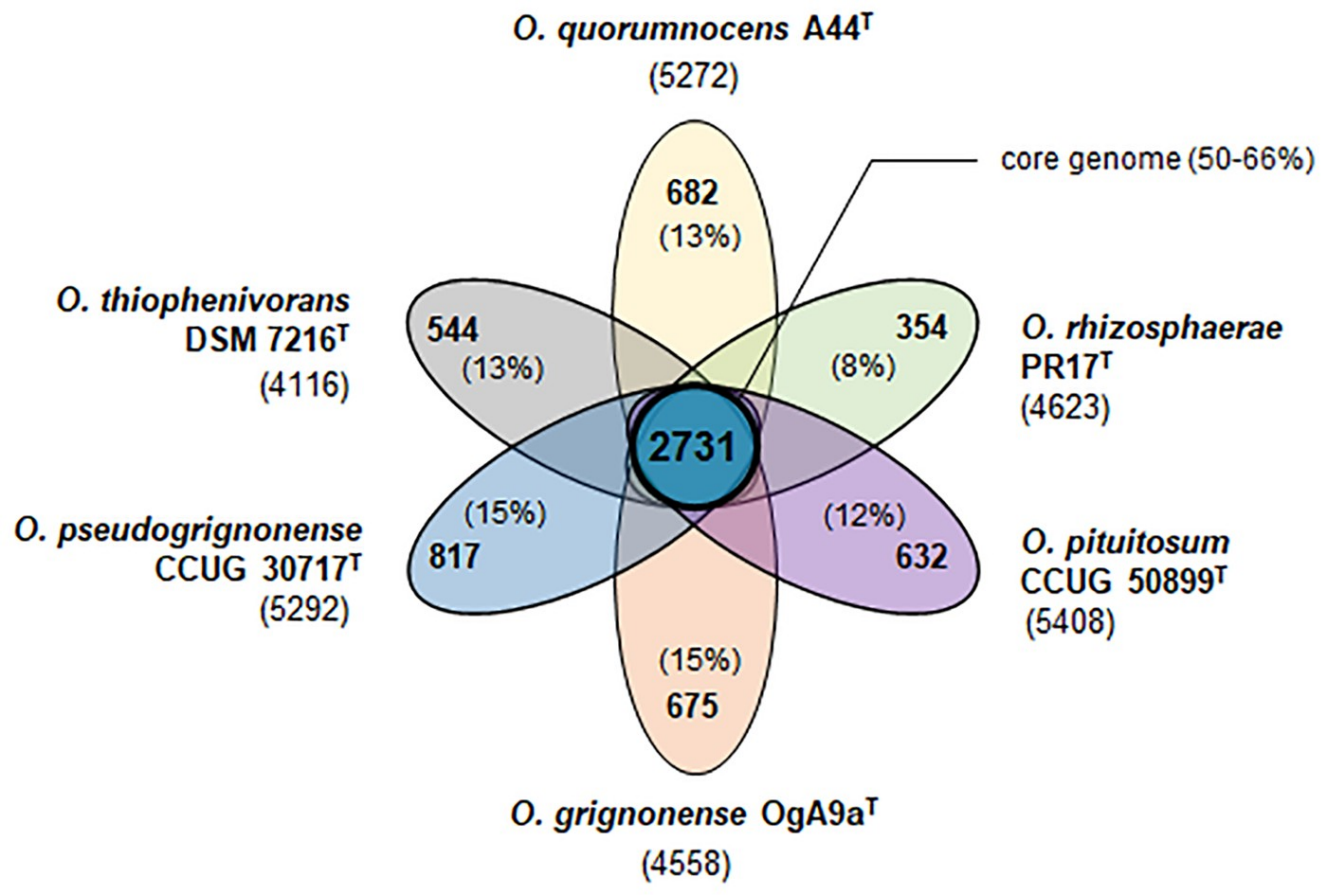
Flower plot depicting the core genome (in the center) and strain-specific genes (in the petals) for the group of six analyzed *Ochrobactrum* spp. type strains. Accession numbers for the sequences can be found in Table 2. Comparative genome analysis was performed using EDGAR. Total number of coding sequences identified by Prokka annotation tool is given next to designations of the particular strains.

Obtaining genome sequences of A44^T^ and the 5 related type strains (*O*. *pituitosum* CCUG 50899^T^, *O*. *rhizosphaerae* PR17^T^, *O*. *grignonense* OgA9a^T^, *O*. *pseudogrignonense* CCUG 30717^T^, and *O*. *thiophenivorans* DSM 7216^T^) enabled us to perform comparative genome analyses using EDGAR [39]. Sequences uniformly annotated with Prokka [40] were used as the input files. Core genome for the six newly sequenced strains consists of 2731 coding sequences, making up 50–66% of each individual genome and 27% of the pan-genome (10296 CDSs) (Fig 2; S5 Table). The set of unique genes harbored by each strain varies from 354 for *O*. *rhizosphaerae* PR17^T^ to 817 for *O*. *pseudogrignonense* CCUG 30717^T^ (8–15%) (S6–S11 Tables—supplementary online content for the amino acid sequences). The remaining genes (32–46%) are shared by a subset of strains. Whereas hypothetical proteins constitute 25% of the core genome, this fraction is significantly enriched within the singletons subsets, ranging from 59% in *O*. *thiophenivorans* DSM 7216^T^ to 80% in *O*. *rhizosphaerae* PR17^T^. The fact that each type strain of the analyzed group contributed new genes to the pan-genome suggests that the pan-genome of *Ochrobactrum* spp. is “open” (as defined by Guimarães *et al*. [41]). However, this hypothesis needs to be confirmed on a broader pool of *Ochrobactrum* spp. genomes, as their availability in public databases continuously increases.

**Fig 3:**
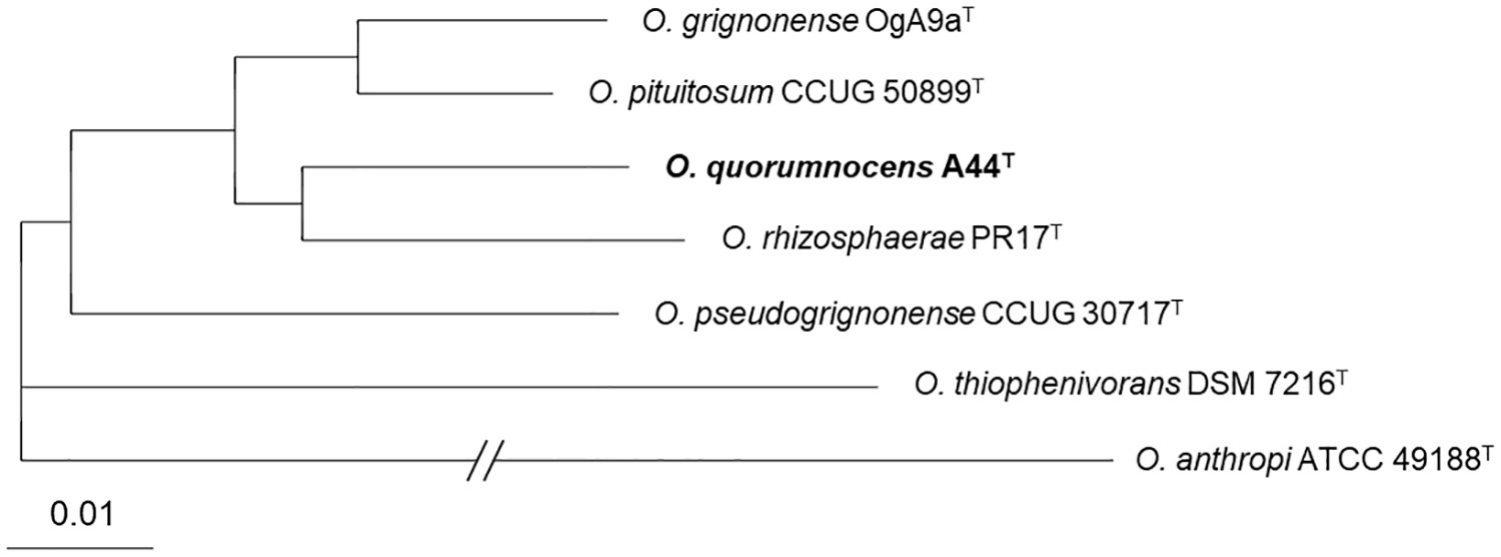
Core genome-based phylogenetic tree for A44^T^ and the related *Ochrobactrum* sp. type strains. The tree was built using EDGAR based 2604 genes per genome (862702 amino acid residues per genome), concatenated to one multiple alignment. The scale indicates phylogenetic distance by substitution events.

Amino acid sequences encoded by 2604 core genes established for A44^T^, its 5 closest relatives, and *O*. *anthropi* ATCC 49188^T^ were used to construct a core genome-based phylogenetic tree. The analysis was performed with EDGAR [40]. According to this approach, grouping of A44^T^, *O*. *rhizosphaerae* PR17^T^, *O*. *grignonense* OgA9a^T^, CCUG 50899^T^, *O*. *pseudogrignonense* CCUG 30717^T^, and *O*. *thiophenivorans* DSM 7216^T^ was analogous to that obtained by MLSA, with *O*. *rhizosphaerae* PR17^T^ indicated as the closest relative of A44^T^ (Fig 3).

### Analysis of chemotaxonomic markers

Sequence-based study was complemented by chemotaxonomic analyses and phenotypic assays. Bacterial fatty acid methyl esters (FAMEs) were obtained according to Bligh and Dyer [42]. Composition of FAMEs obtained for A44^T^, *O*. *pituitosum* CCUG 50899^T^, *O*. *rhizosphaerae* PR17^T^, *O*. *grignonense* OgA9a^T^, *O*. *pseudogrignonense* CCUG 30717^T^, and *O*. *anthropi* ATCC 49188^T^ are shown in S12 Table.

FAME profiles for these closely related species were similar, with small species-specific differences. Major FAMEs produced by A44^T^ were: C_18:1_^9^ (73.3%), C_18:0_ (12.4%), C_16:0_ (7.8%), and, at lower percentage, methyl cis-9,10-methyleneoctadecanoate (4%), C_16:1_^9^ (1.9%), and C_17:0_ (0.6%).

The whole-cell MALDI-TOF mass spectrometry profiles were performed for A44^T^ and the related strains: *O*. *rhizosphaerae* PR17^T^, *O*. *grignonense* OgA9a^T^, *O*. *pituitosum* CCUG 50899^T^, *O*. *pseudogrignonense* CCUG 30717^T^, *O*. *thiophenivorans* DSM 7216^T^, and *O*. *anthropi* ATCC 49188^T^. The tested strains presented similar peak patterns with subtle species-specific differences (S2 Fig). S13 Table summarizes the obtained m/z values and indicates distinctiveness of A44^T^ from the closely related species.

### Biochemical and phenotypic traits

A set of phenotypic assays was performed for A44^T^ and the closely related strains: *O*. *rhizosphaerae* PR17^T^, *O*. *grignonense* OgA9a^T^, *O*. *pituitosum* CCUG 50899^T^, *O*. *pseudogrignonense* CCUG 30717^T^, *O*. *thiophenivorans* DSM 7216^T^. Comparison of biochemical traits, determined with GEN III MicroPlates (Biolog, USA), revealed twelve differences between A44^T^ and *O*. *rhizosphaerae* PR17^T^, twelve between A44^T^ and *O*. *grignonense* OgA9a^T^, fifteen for A44^T^ and *O*. *thiophenivorans* DSM 7216^T^, sixteen for A44^T^ and *O*. *pituitosum* CCUG 50899^T^, and seventeen for A44^T^ and *O*. *pseudogrignonense* CCUG 30717^T^ (S14 Table). Moreover, with the API 20NE test (bioMérieux, France), we determined that A44^T^ is negative for the production of urease, unlike the urease-positive *O*. *thiophenivorans* DSM 7216^T^, and is unable to reduce nitrates to nitrites, as opposed to *O*. *grignonense* OgA9a^T^ and *O*. *pseudogrignonense* CCUG 30717^T^. Within the *Ochrobactrum* genus, urease activity can vary between species and should not be considered as a criterion for genus identification [43]. In this work, strain A44^T^, along with the type strains of *O*. *pseudogrignonense* CCUG 30717^T^, *O*. *rhizosphareae* PR17^T^, *O*. *grignonense* OgA9a^T^, and *O*. *pituitosum* CCUG 50899^T^, was shown to be urease-negative on API strips and in the urea-indole medium. On the contrary, strain *O*. *thiophenivorans* DSM 7216^T^, for which no previous data concerning urease activity had been available, gave a positive reaction in the urease assays (S3 Fig).

Motility of the strain, its closest relatives, and *O*. *anthropi* ATCC 49188^T^, was assessed in glass tubes assay after 4 days incubation at 28°C. Strain A44^T^ was found to be motile under all tested conditions. The same was observed for *O*. *rhizosphaerae* PR17^T^ and *O*. *grignonense* OgA9a^T^, whereas *O*. *pseudogrignonense* CCUG 30717^T^ was motile only in the presence of casamino acids, and *O*. *thiophenivorans* DSM 7216^T^ was immotile irrespective of the tested conditions. *O*. *anthropi* ATCC 49188^T^ was motile only in the presence of glucose (with and without casamino acids) but not in glycerol, unless supplemented with casamino acids (S15 Table).

We verified the growth ability of A44^T^, the five related strains: *O*. *rhizosphaerae* PR17^T^, *O*. *grignonense* OgA9a^T^, *O*. *pituitosum* CCUG 50899^T^, *O*. *pseudogrignonense* CCUG 30717^T^, *O*. *thiophenivorans* DSM 7216^T^, and *O*. *anthropi* ATCC 49188^T^ in a range of temperatures varying from 7 up to 42°C. Following 7 days of incubation on LB agar plates, all tested strains showed growth at 7, 10, 20, 28, and 37°C, but not at 42°C. However, species-specific differences were observed at 37°C, triggering us to perform detailed growth rate comparison in LB for this temperature. Results revealed that the strains isolated from clinical samples, namely *O*. *anthropi* ATCC 49188^T^ and *O*. *pseudogrignonense* CCUG 30717^T^, showed the highest growth rate at 37°C among the tested strains. Strains A44^T^ and *O*. *grignonense* OgA9a^T^ grew well, *O*. *pituitosum* CCUG 50899^T^ and *O*. *thiophenivorans* DSM 7216^T^ grew moderately, and *O*. *rhizosphaerae* PR17^T^ showed weak growth at 37°C (S4 Fig). When grown for 5 days in the LB medium containing varying concentrations of NaCl, all tested strains could grow in medium containing up to 4.5% NaCl (S16 Table). However, the growth of A44^T^, *O*. *pseudogrignonense* CCUG 30717^T^, *O*. *thiophenivorans* DSM 7216^T^, and *O*. *anthropi* ATCC 49188^T^ was less affected by the increasing concentration of NaCl than it was in the case of other investigated strains. Only those four microorganisms showed any growth at 6% of NaCl, with *O*. *anthropi* being the most resistant to salinity. According to the results from GEN III MicroPlates (Biolog, USA), none of the tested strains (*viz*. A44^T^, *O*. *grignonense* OgA9a^T^, *O*. *thiophenivorans* DSM 7216^T^, *O*. *pseudogrignonense* CCUG 30717^T^, *O*. *pituitosum* CCUG 50899^T^, *O*. *rhizosphaerae* PR17^T^) could grow at pH 5, and all strains, apart from *O*. *rhizosphaerae* PR17^T^, could grow at pH 6 (S14 Table).

**Fig 4:**
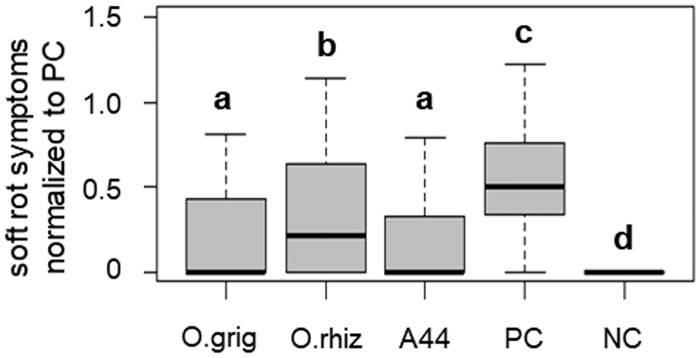
Inactivation of C6-HSL by *O*. *quorumnocens* A44^T^ and the type strains of the related *Ochrobactrum* spp. Error bars in the graph indicate standard deviation values for the mean values of two independent experiments. RLU—relative luminescence of *E*. *coli* [pSB401] biosensor. O. quorum.—*O*. *quorumnocens* A44^T^, O. rhizos.—*O*. *rhizosphaerae* PR17^T^, O. grignon.—*O*. *grignonense* OgA9a^T^, O. pseudogr.—*O*. *pseudogrignonense* CCUG 30717^T^, O. thioph.—*O*. *thiophenivorans* DSM 7216^T^, O. pituit.—*O*. *pituitosum* CCUG 50899^T^, O. anth.—O. *anthropi* ATCC 49188^T^, ref.—reference sample to which no potential C6-HLS-degrading agent was added.

Considering that A44^T^ was previously studied for its ability to inactivate the AHL signal molecules, we verified the potential of the related *Ochrobactrum* sp. type strains to inactivate AHLs. Interestingly, *O*. *rhizosphaerae* PR17^T^ was found to be unable to interfere with quorum sensing. Using a modified assay as described by Jafra and van der Wolf [44] and the AHL biosensor *E*. *coli* pSB401 [45], we showed that *O*. *rhizosphaerae* PR17^T^ cannot inactivate C6-HSL—one of the AHLs efficiently inactivated by A44^T^, *O*. *pituitosum* CCUG 50899^T^, *O*. *grignonense* OgA9a^T^, *O*. *pseudogrignonense* CCUG 30717^T^, and *O*. *thiophenivorans* DSM 7216^T^ (Fig 4).

**Fig 5:**
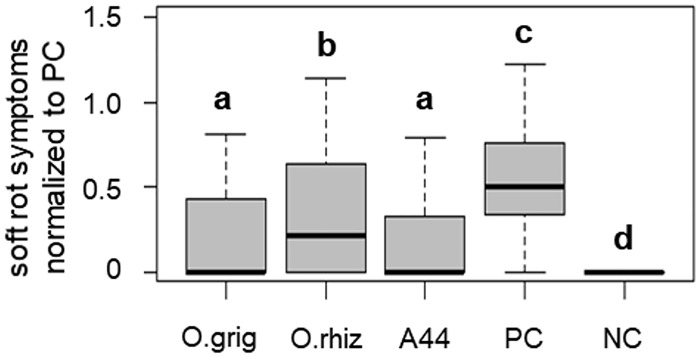
Soft rot symptoms on potato tuber slices inoculated with plant pathogen *P*. *parmentieri* SCC 3193 alone (PC) and co-inoculated with *P*. *parmentieri* and the respective *Ochrobactrum* spp. O. grig—*O*. *grignonense* OgA9a^T^, O. rhiz—*O*. *rhizosphaerae* PR17^T^, and A44 —*O*. *quorumnocens* A44^T^; NC—no pathogen control. In the box plots, the bold lines represent median values, whiskers indicate extreme values and boxes determine the inter-quartile range (Q1–Q3). Groups significantly different (α<0.05) from one another are marked with different letters (a-d).

In line with this finding, strain *O*. *rhizosphaerae* PR17^T^ showed a significantly lower potential to attenuate soft rot caused by *P*. *parmentieri* SCC3193 when compared to A44^T^, as demonstrated in a potato tuber slices assay conducted according to Jafra *et al*. [20] (Fig 5).

List of the selected phenotypic traits which can be used for efficient discrimination between A44^T^ and the related strains is given in Table 2.

**Table 2:** Phenotypic traits for differentiation between *O*. *quorumnocens* sp. nov. and the related *Ochrobactrum* spp. type strains.

Trait	1^A^	2	3	4	5	6
***Carbon source utilization***						
Citric acid	-	+	+	+	+	-
d-maltose	-	+	+	-	-	-
Sucrose	-	+	+	-	-	-
l-rhamnose	+	+	+	+	+	-
l-histidine	+	+/-	+	+	+	-
Pectin	-	+	+	-	-	-
Glucoic acid	+/-	+	+	+	-	+/-
Bromo-succinic acid	+	+	+/-	-	+	+/-
***Other***						
Reduction of nitrates to nitrites	-	-	-	+	+	-
Urease	-	-	-	-	-	+
Inactivation of C6-HSL	+	+	-	+	+	+
Growth at 37°C in LB	good	moderate	weak	good	very good	moderate
Motility	+	+	+	+	+/-^B^	-

^A^ 1—*O*. *quorumnocens* A44^T^, 2—*O*. *pituitosum* CCUG 50899^T^, 3—*O*. *rhizosphaerae* PR17^T^, 4—*O*. *grignonense* OgA9a^T^, 5—*O*. *pseudogrignonense* CCUG 30717^T^, 6—*O*. *thiophenivorans* DSM 7216^T^

^B^ motile in the presence of amino acids, immotile in the absence of amino acids in minimal medium with glycerol as a sole carbon source (details in Supplementary data, S3 Table)

All strains were non-hemolytic on Columbia blood agar (BTL, Poland). Following 19 h of incubation of GEN III plates at 28°C, all strains were negative for the utilization of d-cellobiose, d-raffinose, α-d-lactose, d-melibiose, fusidic acid, d-serine, minocyclin, *p*-hydroxy-phenylacetic acid and did not show growth in the presence of 8% NaCl and at pH 5. In contrast, all strains metabolized α-d-glucose, d-mannose, d-fructose, d-galactose, d-fucose, L-fucose, 1% sodium lactate, d-arabitol, L-alanine, L-glutamic acid, d-galacturonic acid, d-galactonic acid lactone, d-glucuronic acid, and grew in the presence of rifamycin SV, vancomycin, troleandomycin, guanidine HCl, tetrazolium violet, tetrazolium blue, L-lactic acid, L-malic acid, lithium chloride, potassium tellurite, acetoacetic acid, acetic acid, aztreonam.

### Determining the genetic background of distinct urease activity and motility among strains

It is often found in literature that the presented results of genome analyses or genome comparisons are limited to ‘dry’ numeric data sets. To go a small step beyond this scenario, we investigated the genetic background for two phenotypes with differentiating values for the studied group of *Ochrobactrum* spp. strains.

#### Urease-related genes

**Fig 6:**
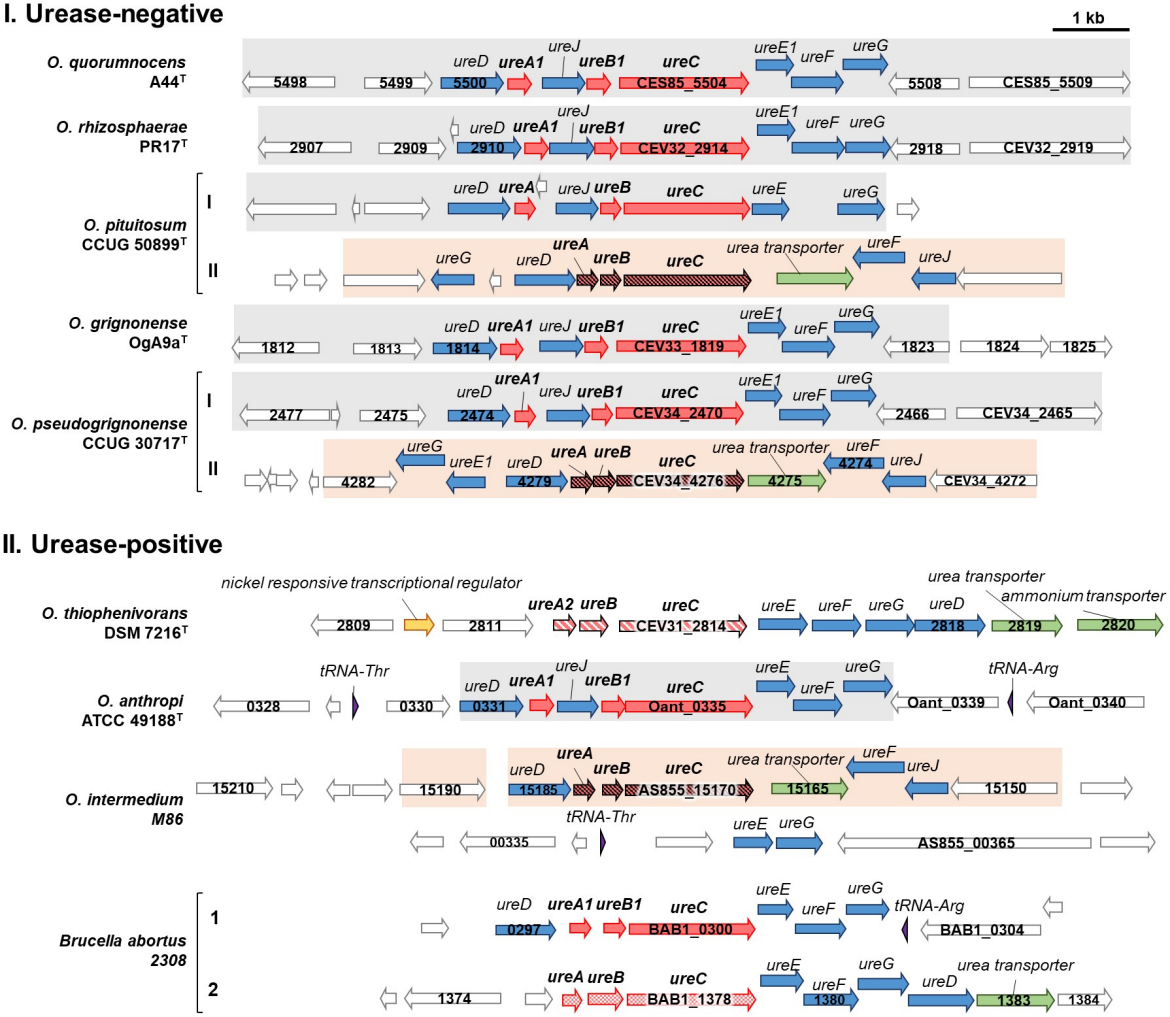
Clusters encoding the homologues of urease genes in *O*. *quorumnocens* A44^T^, the closely related *Ochrobactrum* type strains, and *O*. *anthropi* ATCC 49188^T^, *O*. *intermedium* M86, and *Brucella abortus* 2308. The clusters were divided into two groups according to their origin from urease-negative (I) and urease-positive (II) strains, as determined in a diagnostic urease assay. ORF colors represent: structural genes (red), accessory genes (blue), transporter genes (green), nickel metabolism related genes (yellow), tRNA encoding regions (violet). Differentiating design patterns were used for the structural genes *ureC*, *ureA*, and *ureB* to indicate different/similar alleles. Matching background colors were used for clusters/cluster regions showing structural resemblance. ORF numbers represent the respective loci in the genomes of the analyzed strains.

Ureases are enzymes which catalyze the hydrolysis of urea to ammonia and carbon dioxide. Bacterial ureases are complex nickel-dependent enzymes, the synthesis of which requires three structural genes, *ureABC*, encoding gamma, beta, and alpha subunits, respectively, and four accessory genes, *ureDEFG*, involved in the protein assembly. We investigated the presence and the relative position of the *ureABCDEFG* genes in the six newly-sequenced *Ochrobactrum* spp. genomes. We found that all analyzed strains, irrespective of being urease-positive or -negative, possessed homologues of genes from the *ure* operon. However, the organization and the genomic context of the urease clusters showed high diversity (Fig 6).

Among the urease-negative strains, *O*. *quorumnocens* A44^T^, *O*. *rhizosphaerae* PR17^T^, and *O*. *grignonense* OgA9a^T^ had a single urease cluster (further referred to as type I) with a similar gene organization. In this type of cluster, apart from *ureABCDEFG*, also the *ureJ* gene, encoding transmembrane hydrogenase/urease accessory protein of unclear function, was identified [46]. *O pituitosum* CCUG 50899^T^ and *O*. *pseudogrignonense* CCUG 30717^T^ were found to have two urease clusters—one of type I and a second, distinct operon (type II). The type II cluster had a different organization of genes and contained an urea transporter. For the structural proteins UreA, UreB and UreC, the identity was higher for homologues derived from the same cluster type, although from different strains, than between homologues from different cluster types. For instance the identity of protein homologues for type I clusters from of A44^T^ and *O*. *pseudogrignonense* CCUG 30717^T^ was 98% for UreC and 95% for both UreA and UreB, in comparison to 70%, 71% and 66%, respectively, for the homologues of these proteins from clusters I and II of *O*. *pseudogrignonense*.

We compared the organization of the urease clusters of the *O*. *quorumnocens* A44^T^ and the related strains to that of three urease-positive clinical isolates: *O*. *anthropi* ATCC 49188^T^, *O*. *intermedium* M86, and *Brucella abortus* 2308 (Fig 5). *O*. *anthropi* ATCC 49188^T^ harbors a single cluster resembling that of type I, however settled in a different genetic context. In *O*. *intermedium* M86, single homologues of the *ure* genes could be found as well, as reported earlier by Kulkarni *et al*. [47]. We found that the *ure* operon in *O*. *intermedium* M86 is similar to that of type II from *O*. *pituitosum* CCUG 50899^T^ and *O*. *pseudogrignonense* CCUG 30717^T^, yet deprived of the *ureE* and *ureG* accessory genes. The missing genes could be located in a remote region of the genome. Neither cluster type I nor II of the studied *Ochrobactrum* spp. strains resembled the two urease clusters present in *B*. *abortus* 2308 [48]. Difference between the urease type from *O*. *intermedium* M86 and that of *Brucella* sp. has been previously reported by [49].

*O*. *thiophenivorans* DSM 7216^T^, the only urease positive strain of the close relatives of A44^T^ harbors a single urease cluster, however of an entirely different type than those observed for the other analyzed *Ochrobactrum* spp. (Fig 5). We found that the protein encoded by the *ureA* allele from *O*. *thiophenivorans* DSM 7216^T^, while showing >58% identity to that of other *Ochrobactrum* spp. strains, shows high homology to that of a particular group of saline water-isolated alphaproteobacterial strains: *Pseudoruegeria* sp. SK021 (query coverage 91%, identity 91%), *Martelella mediterranea* (91%, 89%) and *Nitratireductor* sp. OM-1 (91%, 88%) [50]. Further analysis revealed that the nearly 7 kb fragment containing the urease cluster from *O*. *thiophenivorans* (nine genes, from *ureA2* to the ammonium transporter encoding gene), shows 91% query coverage and 78% identity at the nucleotide level with an analogous cluster from *Pseudoruegeria* sp. SK021 (MTBG01000044.1: 6099–12600), isolated from the North Sea sediment [51]. Thus, it is highly possible that the unique, in terms of *Ochrobactrum* spp., urease cluster was obtained by *O*. *thiophenivorans via* horizontal gene transfer.

We may only hypothesize that *O*. *quorumnocens* A44^T^ and the related *Ochrobactrum* spp. are urease-negative despite harboring urease-encoding operons due to the point mutations, frameshifts, or deletions/insertions that have accumulated in the (ancestor) clusters. The activity of urease results in increase in pH due to production of ammonia. For human pathogens such as *Helicobacter pylori*, *Yersinia enterocolitica*, and *Brucella* sp. urease activity increases the survival rate in the acidic environment of the host’s gastric tract [48,51,52]. Analogous function was proposed for ureases produced by clinical isolates of *Ochrobactrum* spp. which, like *H*. *pylori*, can also be found in urease-positive gastric biopsies [47, 53]. Also, the non-pathogenic soil microorganisms including *Rhizobium* spp. [54] produce ureases. Mineralization of nitrogen from urea by the soil microbiota makes it more accessible to the plants and is essential for completing the nitrogen cycle. This includes both the naturally occurring urea and the urea-based fertilizers—the most widely used nitrogen fertilizers in agriculture (http://faostat.fao.org). Gram-negative bacteria can obtain nitrogen both from organic compounds, such as amino acids, and from inorganic ammonia—the decomposition product of urea [55].

#### Flagella-related genes

*O*. *thiophenivorans* DSM 7216^T^ was shown to be non-motile, both in the original study [6] and under the conditions tested in this work. To investigate the genetic background of this phenotype, we searched the genomes of all six strains for genes related to flagella formation [56]. Our results showed that *O*. *thiophenivorans* DSM 7216^T^ lacks 12 out of 25 investigated genes, 11 of which were among the 24 genes present in every other strain (Table 3).

**Table 3:** Flagella-related genes in *O*. *quorumnocens* A44^T^ and the related *Ochrobactrum* spp.

Gene symbol	Gene Name/Annotation	Gene locus in a given strain					
		1^A^	2	3	4	5	6
	flagellar biosynthesis, FliO family protein	CES85_1156	PYSY02000001.1: 1264838 to 1266090 ^B^	CEV32_0466	CEV33_0058	CEV34_3015	CEV31_2471
	flagellin N-methylase family protein	NF	PYSY02000001.1: 1736305 to 1736751	CEV32_0985	CEV33_0518	NF	CEV31_0389
	putative flagellar export protein FliJ	CES85_1827	PYSY02000001.1: 1864435 to 1864827	CEV32_1112	CEV33_0638	CEV34_0632	CEV31_0503
	flgD Ig-like domain protein	NF	NF	NF	NF	CEV34_0645	NF
*fliN*	flagellar motor switch protein FliN	CES85_5570	PYSY02000002.1:1006599 to 1006919	CEV32_2976	CEV33_1682	CEV34_2409	CEV31_4116
	flagellar motor switch FliM family protein	CES85_5572	PYSY02000002.1: 1005195 to 1006133	CEV32_2978	CEV33_1684	CEV34_2407	CEV31_4118
*flgF*	flagellar basal-body rod protein FlgF	CES85_5575	PYSY02000002.1:1002650 to 1003381	CEV32_2981	CEV33_1688	CEV34_2403	CEV31_4122
*fliI*	flagellar protein export ATPase FliI	CES85_5576	PYSY02000002.1:1001304 to 1002646	CEV32_2982	CEV33_1689	CEV34_2402	CEV31_4123
*flgB*	flagellar basal-body rod protein FlgB	CES85_5578	PYSY02000002.1:1000168 to 1000548	CEV32_2984	CEV33_1691	CEV34_2400	CEV31_4125
*flgC*	flagellar basal-body rod protein FlgC	CES85_5579	PYSY02000002.1: 999745 to 1000164	CEV32_2985	CEV33_1692	CEV34_2399	CEV31_4126
	flagellar hook-basal body complex FliE family protein	CES85_5580	PYSY02000002.1: 999410 to 999745	CEV32_2986	CEV33_1693	CEV34_2398	CEV31_4127
*flgG*	flagellar basal-body rod protein FlgG	CES85_5581	PYSY02000002.1:998594 to 999383	CEV32_2987	CEV33_1694	CEV34_2397	CEV31_4128
*flgA*	flagella basal body P-ring formation protein FlgA	CES85_5582	PYSY02000002.1: 998004 to 998501	CEV32_2988	CEV33_1695	CEV34_2396	CEV31_4129
	flagellar basal body-associated FliL family protein	CES85_5586	PYSY02000002.1: 994935 to 995426	CEV32_2992	CEV33_1699	CEV34_2392	NF
*fliP*	flagellar biosynthetic protein FliP	CES85_5587	PYSY02000002.1:994203 to 994938	CEV32_2993	CEV33_1700	CEV34_2391	NF
*fliC*	flagellin	CES85_5588	PYSY02000002.1:993034 to 993930	CEV32_2994	CEV33_1702	CEV34_2390	NF
*fliF*	flagellar M-ring protein FliF	CES85_5590	PYSY02000002.1:991141 to 992886	CEV32_2996	CEV33_1703	CEV34_2389	NF
	flagellar hook-length control FliK family protein	CES85_5594	PYSY02000002.1:986689 to 988022	CEV32_3000	CEV33_1707	CEV34_2385	NF
*flgE*	flagellar hook protein FlgE	CES85_5597	PYSY02000002.1: 983757 to 984959	CEV32_3003	CEV33_1710	CEV34_2382	NF
*flgK*	flagellar hook-associated protein FlgK	CES85_5598	PYSY02000002.1: 982201 to 983655	CEV32_3004	CEV33_1711	CEV34_2381	NF
	bacterial flagellin C-terminal helical region family protein	CES85_5599	PYSY02000002.1: 981149 to 982195	CEV32_3005	CEV33_1712	CEV34_2380	NF
*fliQ*	flagellar biosynthetic protein FliQ	CES85_5603	PYSY02000002.1: 979454 to 979720	CEV32_3009	CEV33_1716	CEV34_2376	NF
*fliR*	flagellar biosynthetic protein FliR	CES85_5605	PYSY02000002.1: 976491 to 977258	CEV32_3011	CEV33_1718	CEV34_2374	NF
	putative flagellar biosynthesis protein FliR	CES85_5606	PYSY02000002.1: 976098 to 976483	CEV32_3012	CEV33_1719	CEV34_2373	NF
	flgN family protein	CES85_5608	PYSY02000002.1: 974784 to 975239	CEV32_3014	CEV33_1722	CEV34_2371	CEV31_3946

^A^ 1—*O*. *quorumnocens* A44^T^, 2—*O*. *pituitosum* CCUG 50899^T^, 3—*O*. *rhizosphaerae* PR17^T^, 4—*O*. *grignonense* OgA9a^T^, 5—*O*. *pseudogrignonense* CCUG 30717^T^, 6—*O*. *thiophenivorans* DSM 7216^T^

^B^ encoding regions in WGS sequence PYSY00000000,

NF—not found,

The missing genes include *fliC* encoding flagellin, *flgE* and *flgK* encoding hook-associated proteins, and *fliF*, *fliQ*, and *fliR* encoding flagellar biosynthetic proteins. In the motile strains, 11 out of the 12 missing genes occur in a cluster, indicating that the loss of motility by *O*. *thiophenivorans* was due to a major deletion of genes essential for flagella formation.

### Formal description of *Ochrobactrum quorumnocens* sp. nov.

*Ochrobactrum quorumnocens* sp. nov. (quo.rum.nocens L. neut. noun, *quorum* quorum; L. present participle *nocens*, from L. verb *noceo* incapacitating, referring to the fact that the type strain was initially studied for its ability to perform *quorum quenching* through inactivation of AHL signal molecules). Cells are Gram-negative, short non-spore-forming rods (1.4 μm in length and 0.9 μm in width), occurring singly and motile by a unipolar flagellum (S5 Fig), urease negative, oxidase and catalase positive, and non-hemolytic on Columbia blood agar. Colonies grown on LB agar are of round shape, smooth surface and edges, low-convex, shiny, and exhibit light beige color. After 5 days of growth at room temperature (22°C) on Tryptic Soy Broth agar, the colonies may become light orange. Growth occurs in 0–4.5% (w/v) NaCl (optimum below 3%), at pH 5.5–10, and temperature range of 7–37°C (optimum around 28°C). The strain grows on McConkey agar but does not ferment lactose. The predominant cellular fatty acid is C_18:1_^9^. In line with the new standards [57], biochemical traits for the formal description of the strain are given in tabular format (S17 Table).

The type strain A44^T^ (= LMG 30544^T^ = PCM 2957^T^) was isolated from the rhizosphere of a potato plant grown in a field in the Netherlands. Genome sequence of the type strain is available from DDBJ/EMBL/GenBank under the accession CP022602.1-CP022605.1. It comprises a main chromosome (2.59 Mbp), a second chromosome (chromid) (2.01 Mbp), and two plasmids (1.03 Mb and 0.02 Mbp). The average G+C content is 53.16%. A44^T^ is able to inactivate AHLs, which are the signal molecules of many Gram-negative bacteria. The formal proposal of the new species *Ochrobactrum quorumnocens* sp. nov. is given in S17 Table with the Taxonumber TA00464 (http://imedea.uib-csic.es/dprotologue/).

## Conclusions

*Ochrobactrum* spp. receive growing interest as members of plant and nematode microbiomes and as potent tools in biotechnology. In this study, on the basis of sequence-based and phenotypic analyses, we propose to establish the QS-interfering strain A44^T^ as a type strain of a novel species: *Ochrobactrum quorumnocens* sp. nov. (Taxonumber TA00464). The strain possesses a multi-replicon genome, characteristic for this genus. Before this study, genomes of only 2 out of 18 *Ochrobactrum* spp. type strains were available. Obtaining draft genomes of 5 of A44^T^-related type strains significantly contributes to the application of golden standard, genome-based approaches in taxonomy of this genus, especially in the light of an increasing number of genome assemblies for new strains, provisionally classified as *Ochrobactrum* sp. To our knowledge, this is the first study in which comparative genome analyses was performed for members of different *Ochrobactrum* species. Moreover, phenotype-to-genotype approach revealed the genetic background of the lack of motility in *O*. *thiophenivorans* DSM 7217^T^ and showed the diversity of urease cluster organization in the studied group of *Ochrobactrum* spp. strains.

## Materials and methods

### Bacterial strains, media and growth conditions

Type strains of *O*. *rhizosphaerae* PR17^T^, *O*. *thiophenivorans* DSM 7216^T^, and *O*. *pituitosum* CCUG 50899^T^ were purchased form Leibniz-Institut DSMZ-Deutsche Sammlung von Mikroorganismen und Zellkulturen GmbH, Germany; strain *O*. *pseudogrignonense* CCUG 30717^T^ was purchased from Culture Collection, University of Göteborg (CCUG), Sweden; strain *O*. *anthropi* ATCC 49188^T^ was purchased form Belgian Co-ordinated Collections of Microorganisms (BCCM/LMG), Belgium; strain *O*. *grignonense* OgA9a^T^ was a kind gift from prof. P. Kämpfer (Justus-Liebig-Universität Gießen, Germany). For routine propagation, all strains were cultured in Miller’s Lysogeny Broth (LB) or on LB solidified with agar (Novagen/Merck, Germany). *Ochrobactrum* spp. strains and *P*. *parmentieri* SCC3191 were grown at 28°C. *Escherichia coli* [pSB401] was grown at 37°C, with the addition of tetracycline (15 μg·mL^-1^). *E*. *coli* [pSB401] was used as a *lux*-based biosensor producing bioluminescence in the presence of AHLs [45]. The ability of *Ochrobactrum* spp. strains to grow at 7, 10, 20, 28, 37, and 42°C for a total of 7 days was assessed on LB agar plates, spot-inoculated with 2 μL of saline suspension of bacterial cells (turbidity adjusted to 0.3 McF). Additionally, the growth rate at 37°C was assessed in the LB medium for a total of 25 h, in 3 biological replicates, 4 technical replicates each. Salt tolerance was determined in LB base (10 g·L^-1^ peptone, 5 g·L^-1^ yeast extract) supplemented with NaCl concentrations ranging from 0 to 9%, in 3 replicates. All growth-related experiments in LB were performed in 96-well format, with orbital shaking (120 rpm) and 200 μl of medium inoculated with 4 μl of cell suspension (0.3 McF) per well. EnVision Multilabel Reader (PerkinElmer, USA) was used to measure OD_600_ of the cultures. The pH growth range of A44^T^ was determined using the PM10 MicroPlate (Biolog, USA), in two replicates.

### 16S rRNA gene analysis and MLSA

Phylogenetic analyses were performed with the use of MEGA 7 software [58]. Dendrograms for the near complete 16S rRNA gene sequences (1335 bp) of A44^T^ and the type strains of all 18 validly published *Ochrobactrum* spp. were obtained using the three clustering algorithms: maximum likelihood based on the Tamura 3-parameter model, neighbor joining, and maximum parsimony. All positions containing gaps and missing data were eliminated. There was a total of 1331 positions in the final dataset [59,60]. Multilocus Sequence Analysis (MLSA) [32] was performed for a concatenated set (3512 bp) of three genes: 16S rRNA gene (1335 bp), *gyrB* (1012 bp), and *groEL* (1165 bp).

### Genome sequencing and genome-based phylogeny

Genome sequencing of *Ochrobactrum* sp. type strains, with the exception of *O*. *pituitosum* CCUG 50899^T^, was performed at BaseClear B.V., the Netherlands. Draft genome of *O*. *pituitosum* CCUG 50899^T^ was obtained by a combined use of Illumina MiniSeq and Oxford Nanopore MinION platforms at IFB UG & MUG, Poland. Sequence of *O*. *pituitosum* CCUG 50899^T^ was automatically annotated using the NCBI Prokaryotic Genome Annotation Pipeline [61]. All other sequences were annotated using the IGS Annotation Engine [33] (Institute for Genome Sciences, University of Maryland School of Medicine, USA). The whole-genome average nucleotide identity (ANIb) was calculated with the use of JSpecies software with default settings [62]. Digital DNA-DNA hybridization was carried out with Genome-to-Genome Distance Calculator 2.0 (GGDC) using the recommended BLAST+ alignment with formula 2 (identities/HSP length) (http://ggcd.dsmz.de/distcalc2.php) [63]. The single species thresholds recommended ANIb and GGDC are 95% [62] and 70% [63], respectively. Analysis of the functional annotation was done using the Kyoto Encyclopedia of Genes and Genomes (KEGG) (https://www.genome.jp/kegg/) [38].

### Comparative genomics

CDS (CoDing Sequences) count for *O*. *pituitosum* CCUG 50899^T^ was obtained using Prokka [40]. Other CDS counts were derived from the respective GenBank files. Comparative genome analysis was performed using EDGAR plaftorm (http://edgar.computational.bio) [39]. The core genome and the singletons for a set of 6 related *Ochrobactrum* spp. strains were calculated for Prokka-annotated genomes using EDGAR (http://edgar.computational.bio).

### Determination of chemotaxonomic markers

Bacterial fatty acids were obtained from the whole bacterial cells grown overnight on the LB medium at 28°C. The fatty acids were extracted according to the method described by Bligh and Dyer, 1959 [42] and subjected to gas chromatography and mass spectrometry (Shimadzu QP 2010 SE system). Identification of FAMEs was performed with respect to standards (Bacterial Acid Methyl Esters, Sigma-Aldrich, USA). For the whole-cell MALDI-TOF mass spectrometry profiles, bacteria were grown overnight on LB agar plates at 28°C. The analysis was performed in ferulic acid (10 mg·mL^-1^) dissolved in 17% formic acid, 33% acetonitrile and 50% water as matrix. Protein mass fingerprints were obtained using the MALDI-TOF/TOF 5800 mass spectrometer (AB Sciex, Framingham, MA, USA), with detection in the linear middle mass (4000–20000 Da), positive ion mode for a total of 1000 laser shots by a 1 kHz OptiBeam laser (YAG, 349 nm). Registered spectra were analyzed with Data Explorer software (AB Sciex).

### Biochemical and phenotypic assays

Biochemical traits and enzymatic activities were determined using GEN III Microplates (Biolog, USA) and API 20NE tests (bioMérieux, France) according to the manufacturer’s protocols, in two independent experiments. The urease activity of the tested strains was also verified, in two biological replicates, using the urease-indole diagnostic broth (BioMaxima, Poland). The results were collected after 24 and 48 hours of incubation at 28°C. The Columbia blood agar (BTL, Poland) was used to assess hemolytic activity. The MacConkey agar from BTL (Poland) was used to determine the growth on this medium. Catalase activity was determined by assessing the generation of oxygen bubbles in the presence of 3% (v/v) hydrogen peroxide. Oxidase activity was tested using plastic strips with a paper zone saturated with *N*,*N*-dimethyl-*p*-phenylenediamine oxolate and α-naphthol (Sigma-Aldrich, USA). LB agar and Tryptic Soy Agar TSA (Thermo Fisher Scientific, USA) were used for determination of colony morphology. Motility was assessed in glass tubes filled with 5 mL of the M9 minimal medium [64] containing 0.3% agar supplemented with 0.4% glycerol or 0.4% glucose, and with or without the addition of 0.5% casamino acids. Media in tubes were stab inoculated and incubated at 28°C for 4 days.

### Cell micrography

Imaging of the A44^T^ cells was performed with the use of atomic force microscope (AFM). Bacteria were grown in the M9 minimal medium supplemented with 0.4% glucose, overnight on a microscopy glass cover slide placed in a Petri dish, with gentle shaking (60 rpm) at 28°C. The slides were washed with water, samples were fixed with 2.5% glutaraldehyde (Sigma Aldrich) for 2 hours, washed again and dried in air. Cells were imaged in air using Bioscope Resolve (Bruker), in ScanAsyst (Peak Force Tapping) mode, with the application of ScanAsyst Air probe (f0 7.0 kHz, diameter <12 nm, k:0.4 N/m).

### AHL inactivation assay

The ability of the tested *Ochrobactrum* sp. type strains to inactivate AHLs was determined in a modified assay described by Jafra and van der Wolf [44]. Briefly, bacterial cells from overnight cultures were harvested and re-suspended in the MOPS-buffered LB, pH 6.5. Turbidity was adjusted to 5 units in McFarland scale (McF) (~3.5×10^8^ cfu·mL^-1^). 50 μl of cell suspension was added to 50 μl of 10 μM C6-HSL (Sigma-Aldrich, USA) in M63 0.12% agar in white/clear bottom 96-well plates [36]. The plates were incubated for 16 h at 28°C, without shaking. The remaining AHLs were detected using *E*. *coli* [pSB401] biosensor [45], emitting light in the presence of C6-HSL.

### Plant protection assay on potato tuber slices

Potato tuber slices assay was performed as previously described [20]. Ware (table) potato tubers of cultivar Gala were obtained in local grocery stores (Gdansk, Poland).

### Genome mining for urease and flagella-related genes

BLAST search tools [65] available from NCBI were used to determine the presence of urease and flagella-related genes in the genomes of A44^T^ and the related *Ochrobactrum* spp. (GenBank accession numbers for genomes given in Table 1). For *O*. *pituitosum*, annotation of clusters A (extracted from PYSY02000003.1) and B (extracted from PYSY02000002.1) was performed with RAST: (http://rast.nmpdr.org/). The CLC Main Workbench 7 (QIAGEN Bioinformatics) and PowerPoint 2016 (Microsoft) were applied to visualize the urease clusters.

## Supporting information

S1 Fig Evolutionary relationship of *Ochrobactrum* spp. based on molecular phylogeny of partial 16S rRNA gene sequence. (A) The phylogenetic tree obtained using the neighbor-joining method. The percentage of replicate trees in which the associated taxa clustered together in the bootstrap test (1000 replicates) are shown next to the branches. The tree is drawn to scale, with branch lengths in the same units as those of the evolutionary distances used to infer the phylogenetic tree. (B) Dendrogram obtained using the maximum parsimony method. Tree #1 out of 2 most parsimonious trees (length = 392) is shown. The consistency index is (0.489051), the retention index is (0.585799), and the composite index is 0.376585 (0.286486) for all sites and parsimony-informative sites (in parentheses). The MP tree was obtained using the Subtree-Pruning-Regrafting (SPR) algorithm with search level 1 in which the initial trees were obtained by the random addition of sequences (10 replicates). For both (A) and (B), the analysis involved 20 nucleotide sequences. All positions containing gaps and missing data were eliminated. There was a total of 1331 positions in the final dataset. The analyses were conducted in MEGA7.
(TIF)

S2 Fig MALDI-TOF profiles for *O*. *quorumnocens* strain A44^T^ and the related *Ochrobactrum* sp. type strains. The analysis was performed in ferulic acid (10 mg·mL^-1^) dissolved in 17% formic acid, 33% acetonitrile, and 50% water as matrix. Protein mass fingerprints were obtained using the MALDI-TOF/TOF 5800 mass spectrometer (AB Sciex, Framingham, MA, USA), with detection in the linear middle mass (4000–20000 Da), positive ion mode for a total of 1000 laser shots by a 1 kHz OptiBeam laser (YAG, 349 nm). Registered spectra were analyzed with Data Explorer software (AB Sciex). All MALDI-TOF MS spectra analyses used in this study were averages of at least four replicated measurements per analyzed strain.
(TIF)

S3 Fig Urease production in the urease-indole medium (BioMaxima, Poland) by *O*. *quorumnocens* A44^T^ (1) and the type strains of *O*. *pituitosum* (2), *O*. *rhizosphaerae* (3), *O*. *grignonense* (4), *O*. *pseudogrignonense* (5), *O*. *thiophenivorans* (6), and *O*. *anthropi* (7) following 24 h (A) and 48 h (B) of incubation at 28°C. (TIF)

S4 Fig Growth of *O*. *quorumnocens* A44^T^ and the related *Ochrobactrum* spp. type strains in LB at 37°C. The experiment was performed in a 96-well format, with the use of EnVision automatic plate reader. Each point represents an average value of 12 measurements, taken in 3 biological replicates, 4 technical replicates each.
(TIF)

S5 Fig Micrographs of *O*. *quorumnocens* A44^T^ taken with atomic force microscope. Cells were imaged in air using Bioscope Resolve (Bruker), in ScanAsyst (Peak Force Tapping) mode, with the application of ScanAsyst Air probe (f_0_ 7.0 kHz, diameter <12 nm, k: 0.4 N/m).
(TIF)

S1 Table 16S rRNA gene matrix. (XLSX)

S2 Table Genomes sequenced in this study in numbers. (XLSX)

S3 Table Average nucleotide identity for pairwise comparison of genome of A44^T^ and all 65 genome assemblies, available in GenBank in April 2018. Analysis was performed using JSpeciesWS. Shown in bold are sequences obtained in this study.
(XLSX)

S4 Table Role category breakdown for the total of 5272 ORFs comprising the genome of *O*. *quorumnocens* sp. nov. A44^T^. (XLSX)

S5 Table List of annotations for proteins in the core genome for a set of 6 related *Ochrobactrum* spp. strains: *O*. *quorumnocens* A44^T^, *O*. *grignonense* OgA9a^T^, *O*. *thiophenivorans* DSM 7216^T^, *O*. *pseudogrignonense* CCUG 30717^T^, *O*. *pituitosum* CCUG 50899^T^, and *O*. *rhizosphaerae* PR17^T^. The core genome was calculated for Prokka-annotated genomes using EDGAR (http://edgar.computational.bio). Genome of *O*. *quorumnocens* was used as a reference.
(XLSX)

S6 Table List of annotations for singletons for *O*. *quorumnocens* A44^T^, calculated with respect to a set of 6 related *Ochrobactrum* spp. strains: *O*. *quorumnocens* A44^T^, *O*. *grignonense* OgA9aT, *O*. *thiophenivorans* DSM 7216^T^, *O*. *pseudogrignonense* CCUG 30717^T^, *O*. *pituitosum* CCUG 50899^T^, and *O*. *rhizosphaerae* PR17^T^. The analysis was performed for Prokka-annotated genomes using EDGAR (http://edgar.computational.bio). Genome of *O*. *quorumnocens* was used as a reference.
(XLSX)

S7 Table List of annotations for singletons for *O*. *rhizosphaerae* PR17^T^, calculated with respect to a set of 6 related *Ochrobactrum* spp. strains: *O*. *quorumnocens* A44^T^, *O*. *grignonense* OgA9aT, *O*. *thiophenivorans* DSM 7216^T^, *O*. *pseudogrignonense* CCUG 30717^T^, *O*. *pituitosum* CCUG 50899^T^, and *O*. *rhizosphaerae* PR17^T^. The analysis was performed for Prokka-annotated genomes using EDGAR (http://edgar.computational.bio). Genome of *O*. *quorumnocens* was used as a reference.
(XLSX)

S8 Table List of annotations for singletons for *O*. *pituitosum* CCUG 50899^T^, calculated with respect to a set of 6 related *Ochrobactrum* spp. strains: *O*. *quorumnocens* A44^T^, *O*. *grignonense* OgA9aT, *O*. *thiophenivorans* DSM 7216^T^, *O*. *pseudogrignonense* CCUG 30717^T^, *O*. *pituitosum* CCUG 50899^T^, and *O*. *rhizosphaerae* PR17^T^. The analysis was performed for Prokka-annotated genomes using EDGAR (http://edgar.computational.bio). Genome of *O*. *quorumnocens* was used as a reference.
(XLSX)

S9 Table List of annotations for singletons for *O*. *grignonense* OgA9a^T^, calculated with respect to a set of 6 related *Ochrobactrum* spp. strains: *O*. *quorumnocens* A44^T^, *O*. *grignonense* OgA9aT, *O*. *thiophenivorans* DSM 7216^T^, *O*. *pseudogrignonense* CCUG 30717^T^, *O*. *pituitosum* CCUG 50899^T^, and *O*. *rhizosphaerae* PR17^T^. The analysis was performed for Prokka-annotated genomes using EDGAR (http://edgar.computational.bio). Genome of *O*. *quorumnocens* was used as a reference.
(XLSX)

S10 Table List of annotations for singletons for *O*. *pseudogrignonense* CCUG30717^T^, calculated with respect to a set of 6 related *Ochrobactrum* spp. strains: *O*. *quorumnocens* A44^T^, *O*. *grignonense* OgA9aT, *O*. *thiophenivorans* DSM 7216^T^, *O*. *pseudogrignonense* CCUG 30717^T^, *O*. *pituitosum* CCUG 50899^T^, and *O*. *rhizosphaerae* PR17^T^. The analysis was performed for Prokka-annotated genomes using EDGAR (http://edgar.computational.bio). Genome of *O*. *quorumnocens* was used as a reference.
(XLSX)

S11 Table List of annotations for singletons for *O*. *thiophenivorans* DSM7216^T^, calculated with respect to a set of 6 related *Ochrobactrum* spp. strains: *O*. *quorumnocens* A44^T^, *O*. *grignonense* OgA9aT, *O*. *thiophenivorans* DSM 7216^T^, *O*. *pseudogrignonense* CCUG 30717^T^, *O*. *pituitosum* CCUG 50899^T^, and *O*. *rhizosphaerae* PR17^T^. The analysis was performed for Prokka-annotated genomes using EDGAR (http://edgar.computational.bio). Genome of *O*. *quorumnocens* was used as a reference.
(XLSX)

S12 Table Whole-cell fatty acid methyl ester profiles of *Ochrobactrum quorumnocens* sp. nov. and the type strains of the related species. (XLSX)

S13 Table Repeatable markers in the number of ions in the mass to charge (m/z) range of 4000–20000 for *O*. *quorumnocens* sp. nov. A44^T^ and six other *Ochrobactrum* spp. type strains. (XLSX)

S14 Table Biochemical traits of *O*. *quorumnocens* A44^T^ and the related *Ochrobactrum* spp. type strains determined using the Biolog Gen III microplates. Data was collected following 19 h of incubation at 28°C.
(XLSX)

S15 Table Motility of A44^T^ and the related *Ochrobactrum* spp. type strains in the M9 medium. (XLSX)

S16 Table Influence of the NaCl concentration on the final optical density (λ = 580 nm) of bacterial cultures grown for 5 days at 28°C on the LB medium. (XLSX)

S17 Table Description of *Ochrobactrum quorumnocens* sp. nov. according to Digital Protologue TA00464 assigned by the www.imedea.uib.es/dprotologue. (XLSX)
