# Correction: *Ochrobactrum quorumnocens* sp. nov., a quorum quenching bacterium from the potato rhizosphere, and comparative genome analysis with related type strains

**DOI:** 10.1371/journal.pone.0213871

**Published:** 2019-03-11

**Authors:** Dorota M. Krzyżanowska, Tomasz Maciąg, Adam Ossowicki, Magdalena Rajewska, Zbigniew Kaczyński, Małgorzata Czerwicka, Łukasz Rąbalski, Paulina Czaplewska, Sylwia Jafra

The image for Fig 4, “Inactivation of C6-HSL by *O*. *quorumnocens* A44^T^ and the type strains of the related *Ochrobactrum* spp.,” is incorrect. Please see the complete, correct Fig 4 here.

**Fig 4:**
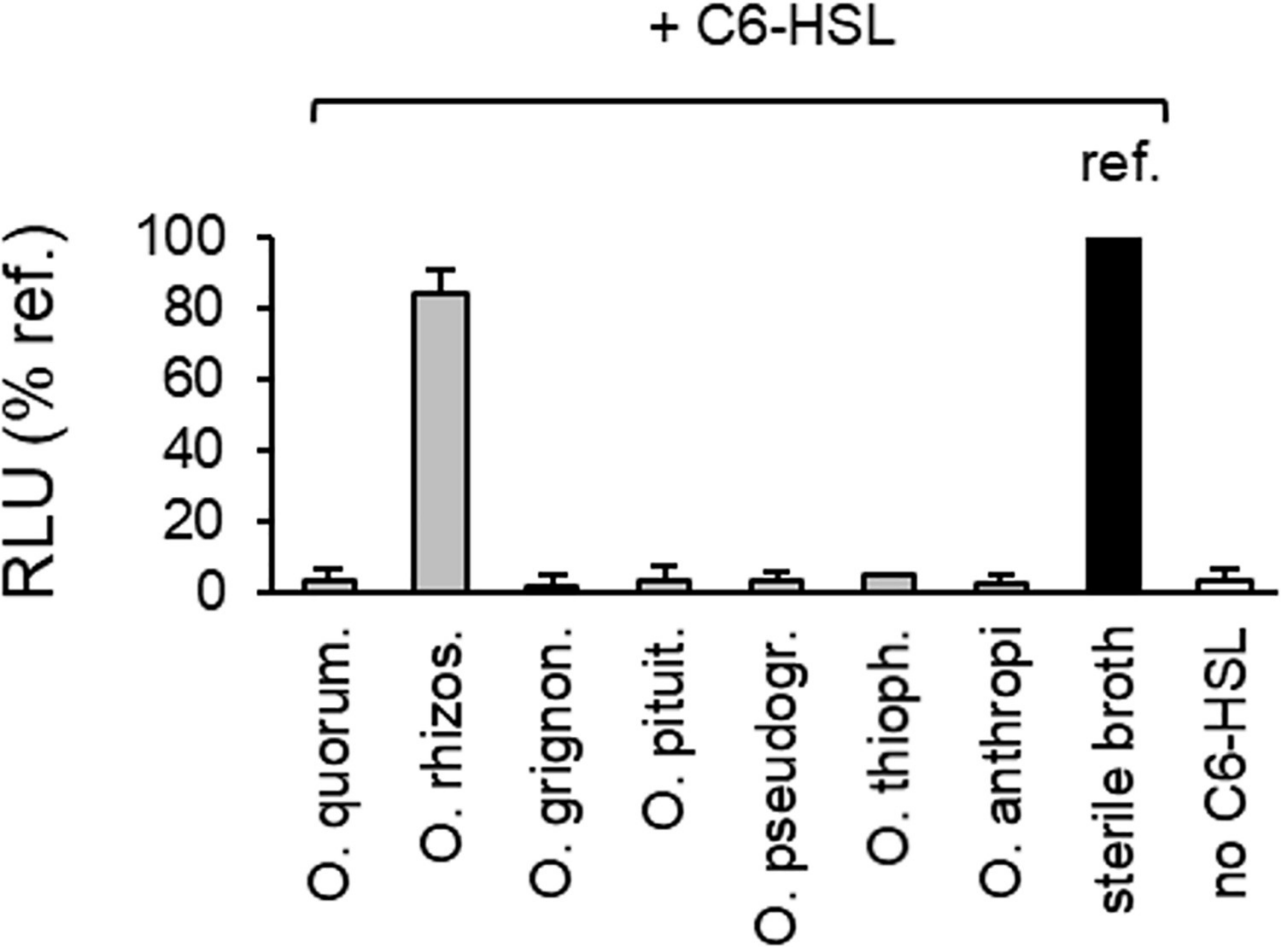
Inactivation of C6-HSL by *O*. *quorumnocens* A44^T^ and the type strains of the related *Ochrobactrum* spp. Error bars in the graph indicate standard deviation values for the mean values of two independent experiments. RLU—relative luminescence of *E*. *coli* [pSB401] biosensor. O. quorum.—*O*. *quorumnocens* A44^T^, O. rhizos.—*O*. *rhizosphaerae* PR17^T^, O. grignon.—*O*. *grignonense* OgA9a^T^, O. pseudogr.—*O*. *pseudogrignonense* CCUG 30717^T^, O. thioph.—*O*. *thiophenivorans* DSM 7216^T^, O. pituit.—*O*. *pituitosum* CCUG 50899^T^, O. anth.—*O*. *anthropi* ATCC 49188^T^, ref.—reference sample to which no potential C6-HLS-degrading agent was added.
