# Supplementary figures and images for: Ochrobactrum quorumnocens sp. nov., a quorum quenching bacterium from the potato rhizosphere, and comparative genome analysis with related type strains
Source: PLoS One. 2019 Jan 22;14(1):e0210874. doi: 10.1371/journal.pone.0210874 (PMC6342446; doi:10.1371/journal.pone.0210874)

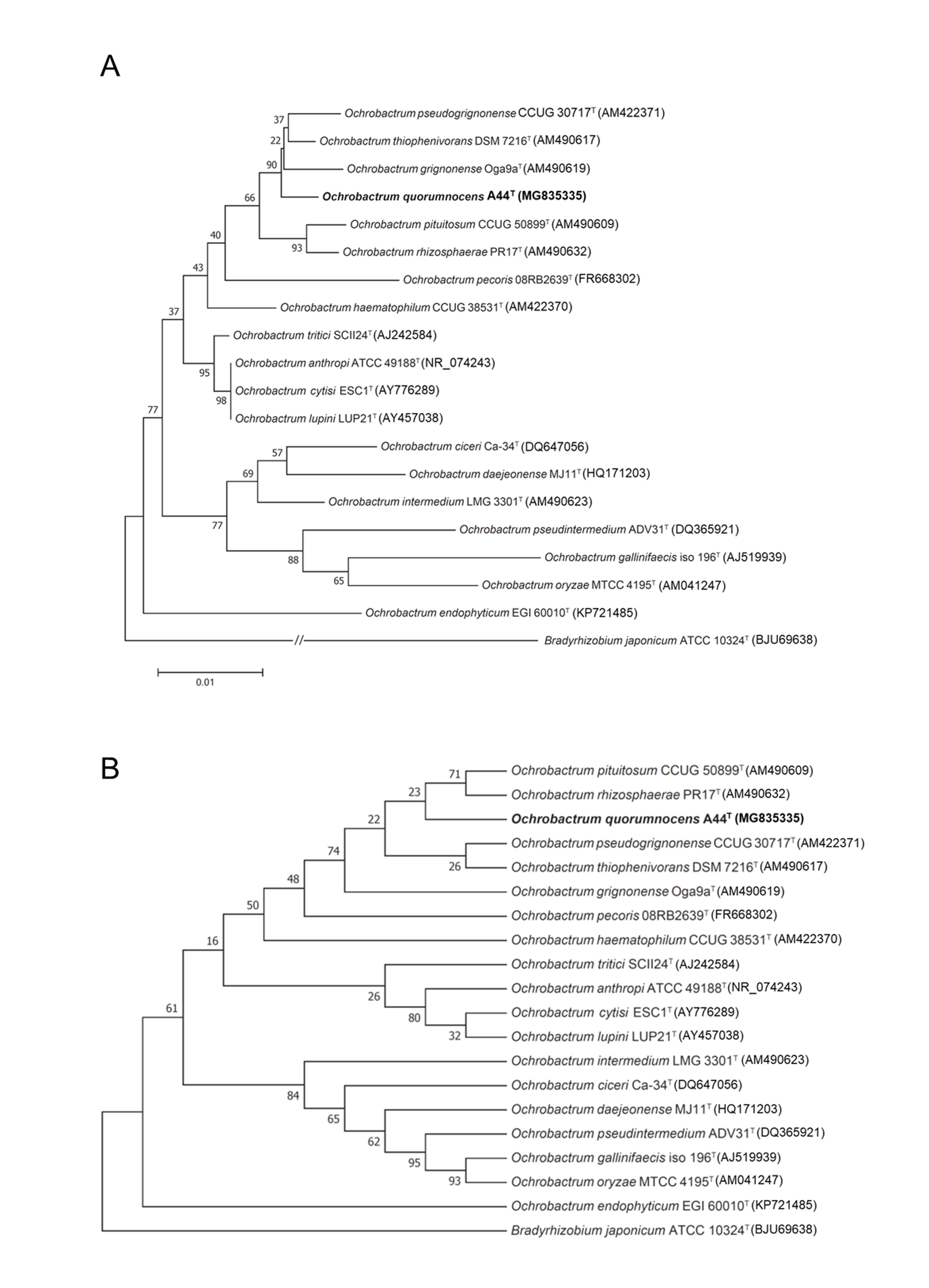

Supplement: S1 Fig — (A) The phylogenetic tree obtained using the neighbor-joining method. The percentage of replicate trees in which the associated taxa clustered together in the bootstrap test (1000 replicates) are shown next to the branches. The tree is drawn to scale, with branch lengths in the same units as those of the evolutionary distances used to infer the phylogenetic tree. (B) Dendrogram obtained using the maximum parsimony method. Tree #1 out of 2 most parsimonious trees (length = 392) is shown. The consistency index is (0.489051), the retention index is (0.585799), and the composite index is 0.376585 (0.286486) for all sites and parsimony-informative sites (in parentheses). The MP tree was obtained using the Subtree-Pruning-Regrafting (SPR) algorithm with search level 1 in which the initial trees were obtained by the random addition of sequences (10 replicates). For both (A) and (B), the analysis involved 20 nucleotide sequences. All positions containing gaps and missing data were eliminated. There was a total of 1331 positions in the final dataset. The analyses were conducted in MEGA7. (TIF) [file pone.0210874.s001.tif]

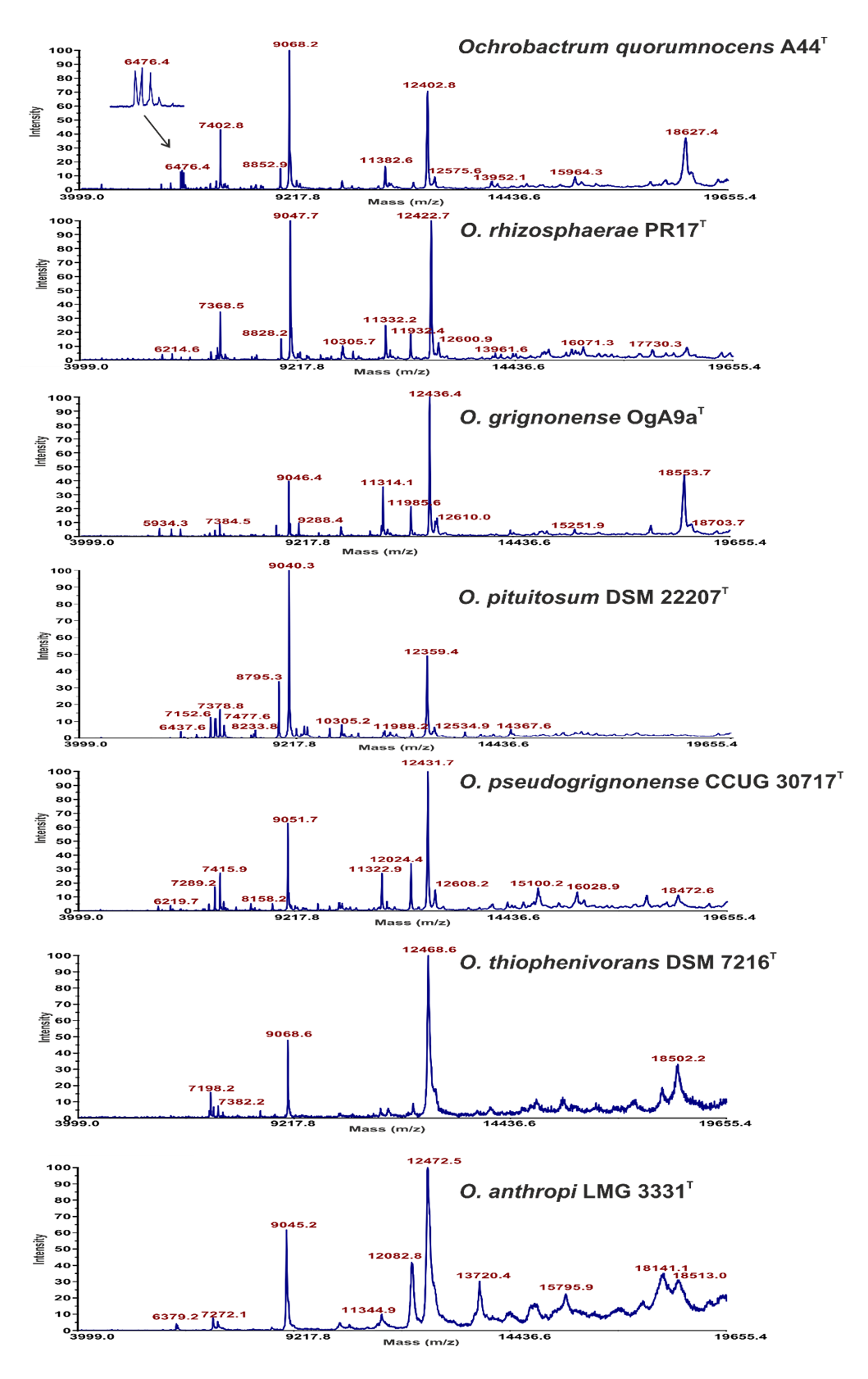

Supplement: S2 Fig — The analysis was performed in ferulic acid (10 mg·mL-1) dissolved in 17% formic acid, 33% acetonitrile, and 50% water as matrix. Protein mass fingerprints were obtained using the MALDI-TOF/TOF 5800 mass spectrometer (AB Sciex, Framingham, MA, USA), with detection in the linear middle mass (4000–20000 Da), positive ion mode for a total of 1000 laser shots by a 1 kHz OptiBeam laser (YAG, 349 nm). Registered spectra were analyzed with Data Explorer software (AB Sciex). All MALDI-TOF MS spectra analyses used in this study were averages of at least four replicated measurements per analyzed strain. (TIF) [file pone.0210874.s002.tif]

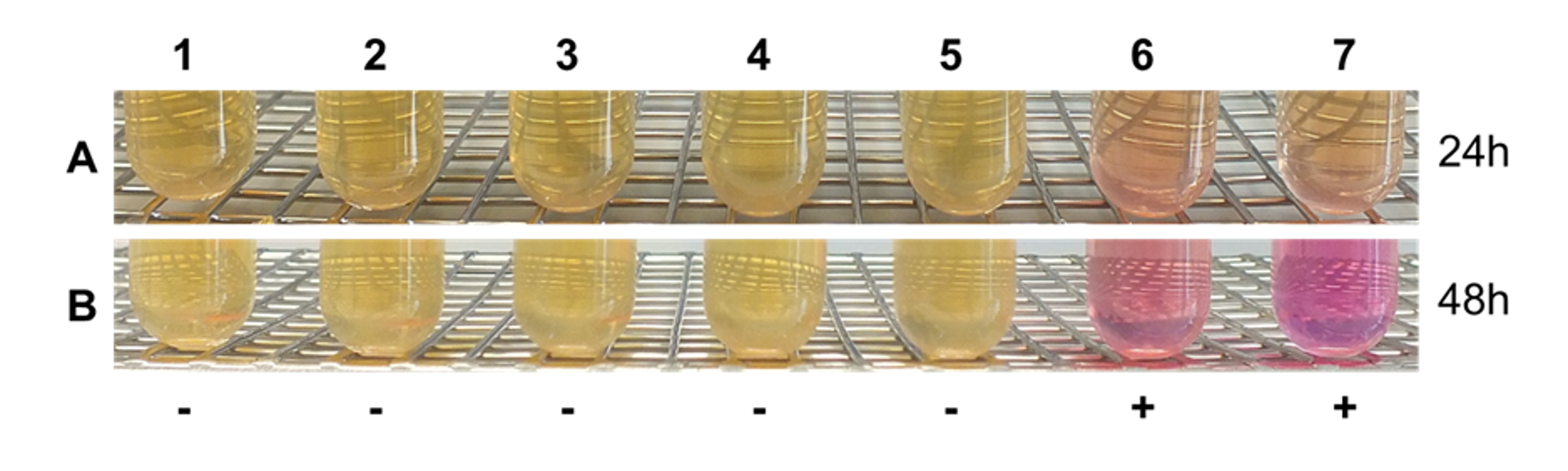

Supplement: S3 Fig — (TIF) [file pone.0210874.s003.tif]

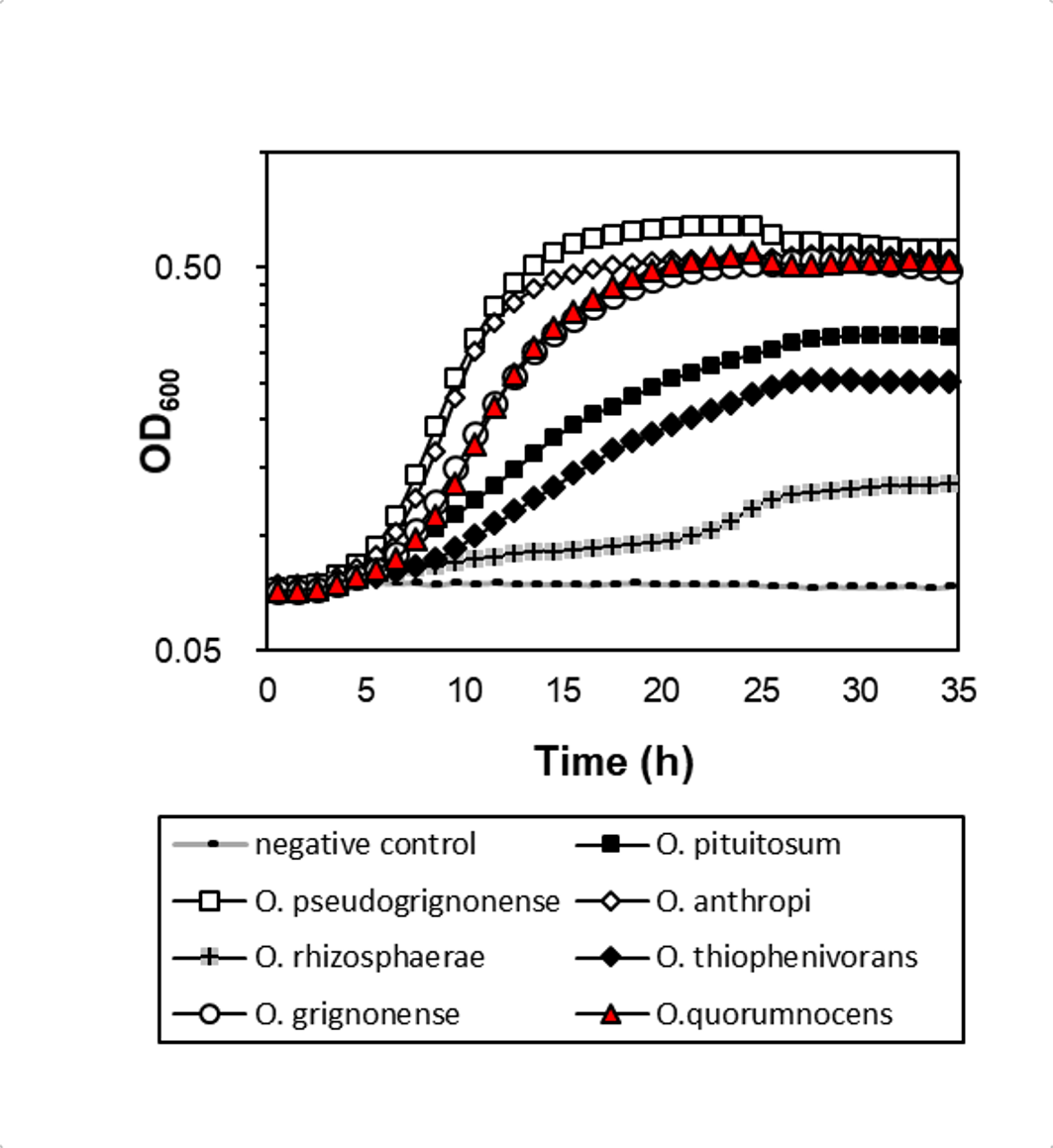

Supplement: S4 Fig — The experiment was performed in a 96-well format, with the use of EnVision automatic plate reader. Each point represents an average value of 12 measurements, taken in 3 biological replicates, 4 technical replicates each. (TIF) [file pone.0210874.s004.TIF]

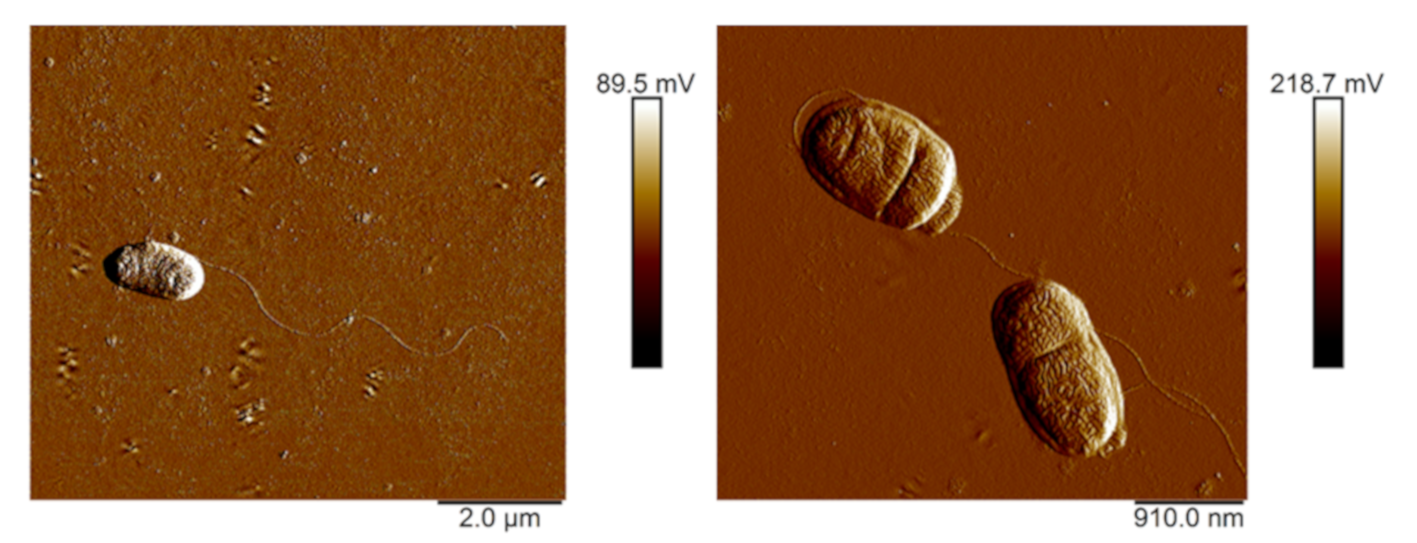

Supplement: S5 Fig — Cells were imaged in air using Bioscope Resolve (Bruker), in ScanAsyst (Peak Force Tapping) mode, with the application of ScanAsyst Air probe (f0 7.0 kHz, diameter <12 nm, k: 0.4 N/m). (TIF) [file pone.0210874.s005.tif]
